# In Situ One‐Pot Synthesis of MOF–Polydopamine Hybrid Nanogels with Enhanced Photothermal Effect for Targeted Cancer Therapy

**DOI:** 10.1002/advs.201800287

**Published:** 2018-04-14

**Authors:** Dongdong Wang, Huihui Wu, Jiajia Zhou, Pengping Xu, Changlai Wang, Ruohong Shi, Haibao Wang, Hui Wang, Zhen Guo, Qianwang Chen

**Affiliations:** ^1^ Hefei National Laboratory for Physical Sciences at Microscale Department of Materials Science and Engineering University of Science and Technology of China Hefei AH 230026 P. R. China; ^2^ High Magnetic Field Laboratory Hefei Institutes of Physical Science Chinese Academy of Sciences The Anhui Key Laboratory of Condensed Matter Physics at Extreme Conditions Hefei AH 230031 P. R. China; ^3^ School of Life Sciences University of Science and Technology of China Hefei AH 230026 P. R. China; ^4^ Department of Radiology First Affiliated Hospital of Anhui Medical University Hefei AH 230022 P. R. China

**Keywords:** cancer theranostics, metal‐organic framework–polymer, multimodal imaging, nanogels, photothermal effects

## Abstract

Herein, a simple one‐pot way is designed to prepare a type of multifunctional metal–organic framework (MOF)‐based hybrid nanogels by in situ hybridization of dopamine monomer in the skeleton of MnCo. The resultant hybrid nanoparticles (named as MCP) show enhanced photothermal conversion efficiency in comparison with pure polydopamine or MnCo nanoparticles (NPs) synthesized under a similar method and, therefore, show great potential for photothermal therapy (PTT) in vivo. The MCP NPs are expected to possess *T*_1_ positive magnetic resonance imaging ability due to the high‐spin Mn‐N6 (*S* = 5/2) in the skeleton of MnCo. To improve the therapy efficiency as a PTT agent, the MCP NPs are further modified with functional polyethylene glycol (PEG) and thiol terminal cyclic arginine–glycine–aspartic acid peptide, respectively: the first one is to increase the stability, biocompatibility, and blood circulation time of MCP NPs in vivo; the second one is to increase the tumor accumulation of MCP‐PEG NPs and improve their therapeutic efficiency as photothermal agent.

Multifunctional hybrid nanogels by combining nanomaterials in a polymer network have attracted much attention in biomedical areas due to their great potential for simultaneous bioimaging, biosensing, and cancer therapy.^1^ Nowadays, many kinds of nanomaterials have been used to prepare hybrid nanogels, including silica, carbon dots/carbon nanotube, graphene/graphene oxide, semiconductor quantum dots, noble metal, and metal/metal oxide.^2^ Recently, nanoscale metal–organic frameworks (MOFs), composed of metal ions or clusters linked by organic bridging ligands, are becoming attractive materials for the synthesis of hybrid nanogels due to their tunable pore space and multiple imaging function. As a subclass of MOFs, Prussian blue analogs (PBAs) with a simple cubic M[M′(C≡N)_6_] framework have been widely explored because of their simple synthesis process, mild reaction conditions, and multifunctional intrinsic properties.^3^ Mn_3_[Co(CN)_6_]_2_ (MnCo), a type of PBAs, has a well‐known double‐perovskite framework, in which the —C≡N— linkers are ordered to create high‐spin Mn–N_6_ (*S* = 5/2) octahedra for excellent magnetic resonance (MR) contrast agents.^4^ In addition, the polarizable π‐electron clouds in cyanide bridges of PBAs could be used to improve the loading capacity of anticancer drug with abundant conjugated structure.^5^ However, the potential toxicity and poor biocompatibility of MnCo limit their biomedical applications in vivo. The combination of MnCo with polymer is an alternative technology to improve their in vivo behavior.

Photothermal therapy (PTT), which utilizes NIR laser–induced thermal ablation of cancer cells, has been extensively explored and found to be a promising alternative approach for future cancer and other disease treatments.^6^ During the past decades, much attention has been paid on the development of NIR laser–assisted PTT agents.^7^ However, commonly used photothermal agents are currently inorganic nanomaterials, such as various gold nanostructures,^8^ copper sulfide nanoparticles,^9^ and carbon nanomaterials,^10^ which may cause long‐term toxicity concerns in their further clinical implementation. Recently, significant attention has been paid to the development of NIR‐absorbing organic materials as PTT agents such as small organic dye (porphysomes, indocyanine green, and heptamenthine)^11^ and polymeric nanoparticles (NPs) (polypyrrole and polyaniline).^12^ Among which, polydopamine (PDA) NPs may be a good candidate due to their good biodegradability and negligible long‐term toxicity since the first preparation by the oxidative polymerization of dopamine in a mild conditions in 2007 by Messersmith and co‐workers.^13^ Meanwhile, it is found that PDA NPs have great potential as a perfect PTT agent for cancer therapy due to their strong NIR absorption for photothermal conversion compared with other inorganic and organic PPT agents.^14^, ^15^ Therefore, it is very meaningful to synthesize MnCo/PDA hybrid nanogels.^16^ So far, the synthesis of MOF‐based hybrid nanogels requires multistep processes including i) synthesis of MOFs and removal of solvents from the pores, ii) incorporation of target polymer molecules into the pore or onto the surface of MOFs, and iii) controlling the polymerization of polymer for preparation of the MOF–polymer hybrid nanogels. Such a complex process is often costly and produces large amounts of waste, which limits their scalable production and reproducibility. More importantly, the reported MOF‐based hybrid nanogels only demonstrated combined function from MOF NPs and polymer network; no synergistic enhanced properties are observed after surface modification of MOFs with polymer.^17^

Considering abundant π‐conjugated structures in PDA NPs, it wonders whether dopamines can be embedded into the skeleton of MnCo through π–π stacking interactions with organic —C≡N— linkers. The resultant MnCo–PDA complex NPs after self‐polymerization of dopamine in the MnCo may have stronger NIR absorbance than single‐component NPs due to the enhanced electron density in complex NPs, leading to the improvement of photothermal conversion ability. Herein, we design a simple one‐pot way to prepare a type of multifunctional MOF‐based hybrid nanogels by in situ hybridization of dopamine monomer in the skeleton of MnCo. The resultant hybrid NPs (named as MCP) show enhanced photothermal conversion efficiency in comparison with pure PDA or MnCo NPs synthesized under a similar method and, therefore, show great potential for PTT in vivo. The MCP NPs are expected to possess *T*_1_ positive MR imaging (MRI) ability due to the high‐spin Mn–N_6_ (*S* = 5/2) in the skeleton of MnCo. To improve the therapy efficiency as a PTT agent, the MCP NPs are further modified with functional polyethylene glycol (PEG) and thiol terminal cyclic arginine–glycine–aspartic acid (cRGD‐SH) peptide, respectively: the first one is to increase the stability and biocompatibility and blood circulation time of MCP NPs in vivo; the second one is to increase the tumor accumulation of MCP‐PEG NPs and improve their therapeutic efficiency as a photothermal agent.^18^

**Figure** **1**a demonstrates the process of synthesis and surface modifications of MCP NPs. The preparation of the MCP NPs is composed of two stages which can be achieved in a one‐pot process: one is that metal ions (Mn^2+^) in manganese acetate and dopamine monomer with phenolic hydroxyl groups can form coordination complex by the chelation,^19^ which further forms MnCo by the assembly of the metal ions and organic linkers (K_3_[Co(CN)_6_]). The dopamine monomer will be encapsulated in the pores of MnCo due to the π–π stacking interaction between organic linkers and monomer, resulting in the formation of a hierarchical MnCo–dopamine intermediate. The other one is the in situ gradual polymerization of dopamine monomers in the porous channels of MnCo MOFs, leading to the formation of MCP NPs. The color change of reaction system from transparent to brown, and then to black indicates the successful polymerization of dopamine monomer (Figure S1, Supporting Information). SCM–PEG–Mal (SCM: succinimide; and Mal: maleimide) is selected as hetero‐bifunctional molecules to perform the PEGylation of MCP due to the abundant —NH_2_ (from PDA) on the surface of MCP NPs. The formed MCP–PEG–Mal can further react with cRGD‐SH, leading to the formation of tumor‐targeted MCP–PEG–RGD NPs.

**Figure 1:**
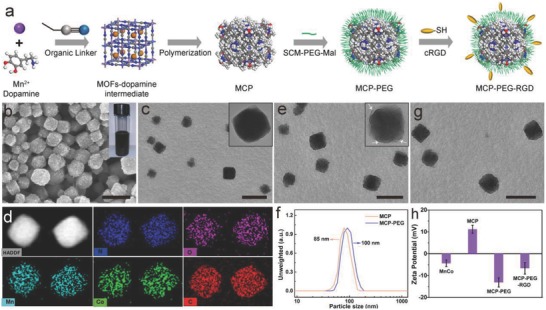
Synthesis and structure and surface modification of MCP NPs. a) The schematic representation of the preparation of MCP, MCP‐PEG, and MCP‐PEG‐RGD NPs. b,c) Scanning electron microscopy (SEM) and TEM images of MCP NPs. The inset in panel (b) is a photograph of aqueous MCP dispersion in a vial. d) HAADF‐STEM image and energy‐dispersive X‐ray (EDX) elemental mapping of MCP NPs. e,g) TEM images of MCP‐PEG and MCP‐PEG‐RGD NPs, respectively. f) Hydrodynamic size distribution of MCP and MCP‐PEG. h) Zeta potential of MnCo, MCP, MCP‐PEG, and MCP‐PEG‐RGD NPs. All scale bars are 100 nm.

The crystalline nature of the as‐prepared MnCo, MOF–dopamine intermediate, and MCP NPs was confirmed by powder X‐ray diffraction pattern (Figures S2 and S3, Supporting Information). The as‐prepared three samples exhibit the same main diffraction peaks, which can be indexed well to the face‐centered cubic phase (JCPDS 89‐3735). Different from MnCo and MOF–dopamine intermediate, a small broad peak centered at 20° is observed in the MCP NPs that should be assigned to amorphous PDA in the pores. Figure 1b,c shows that the as‐obtained MCP NPs have a good monodispersity with a size distribution of 85 nm. Compared with irregular PDA and clear cubic MnCo (Figures S3 and S4, Supporting Information), the rough surface of MCP NPs indicates that the dopamine monomer had been polymerized into the pores and surface of MnCo. In addition, both the Brunauer–Emmett–Teller surface area and pore volume of MCP NPs (30.2 m^2^ g^−1^ and 0.072 cm^3^ g^−1^) are significantly lower than that of MnCo (751.3 m^2^ g^−1^ and 0.42 cm^3^ g^−1^) (Figure S5, Supporting Information), suggesting the occupancy of pores in MnCo. Moreover, the disappearance of pore size at 7.37 nm of MCP compared with pure MnCo also indicated the successful hybridization of polydopamine in the pores of MnCo MOFs.

The chemical composition of MnCo and MCP NPs was determined by high‐angle annular dark‐field scanning transmission electron microscopy (HAADF‐STEM). Evidently, the uniform O element distribution in single MCP NP structure (Figure 1d) suggests the homogeneously hybridization of dopamine monomer in the MnCo. The weight ratio of MnCo/PDA in single MCP NP is 76/24 based on the thermogravimetric analysis (Figure S6 and Table S1, Supporting Information). X‐ray photoelectron spectroscopy analysis of MCP NPs shows two characteristic peaks at 654.1 and 642.0 eV which belong to Mn(II) 2p_1/2_ and Mn(II) 2p_3/2_ spin–orbit peaks respectively (Figure S7, Supporting Information). Besides, a satellite peak around 647.0 eV is observed, further evidencing the existence of Mn(II). The existence of Mn(II) (with five unpaired 3d electron) makes MCP a good candidate to serve as a *T*_1_ contrast agent for MR imaging.

The PEG modification of MCP NPs was confirmed by transmission electron microscopy (TEM) and dynamic light scattering, respectively. As shown in Figure 1e and its inset, the MCP‐PEG NPs are observed to be a slightly rougher than MCP NPs due to the existence of amorphous PEG chains. Meanwhile, the diameter distribution (≈100 nm) of MCP‐PEG NPs is slightly larger than that of MCP NPs because of the presence of PEG chains (Figure 1f). Importantly, the MCP‐PEG NPs showed good dispersity in phosphate‐buffered saline (PBS), Dulbecco's modified Eagle medium, and bovine serum albumin solutions and long‐term aqueous stability in 8× saline solution even being stored for one year without any detectable agglomeration (Figures S8 and S9, Supporting Information). Although the MCP‐PEG‐RGD NPs demonstrate a similar morphology with MCP‐PEG NPs (Figure 1g), the varied zeta potential on the MCP‐PEG (−13.1 ± 1.2 mV) and MCP‐PEG‐RGD (−6.6 ± 2.6 mV) NPs still reveals that RGD molecules had been immobilized onto the surface of MCP‐PEG NPs (Figure 1h).

**Figure** **2**a manifests the typical UV–vis–NIR absorption spectra of the MnCo, PDA, and MCP NPs. While the MnCo has no significant absorption at wavelength NIR region, the MCP and free PDA NPs show a similar absorption peak centered from 500 to 800 nm, indicating that the PDA has been successfully immobilized into the MOF. Moreover, a new adsorption from 550–700 nm has been found. Such vis–NIR adsorption should come from π–π stacking interactions between the polarizable π‐electron clouds of the —C≡N— linkers and PDA, which is similar to the produced NIR adsorption by the interaction between cisplatin and PDA NPs.^20^ Therefore, it is expected that the MCP NPs have a great potential as an excellent photothermal agent due to their strong NIR adsorption. The IR thermal imaging photographs and temperature curves in Figure 2b,c show that the MCP solutions with different concentrations manifested a rapidly temperature increase after being irradiated using a NIR laser (808 nm, 1.0 W cm^−2^). Upon 5 min NIR irradiation, the temperature of the MCP solution (0.05 mg mL^−1^) is raised by 19 °C. As a comparison, the temperature change of water (control) was much less significant (2.5 °C) under the same irradiation conditions, which confirms the photothermal effect of MCP NPs while laser only causes minimal thermal effect.

**Figure 2:**
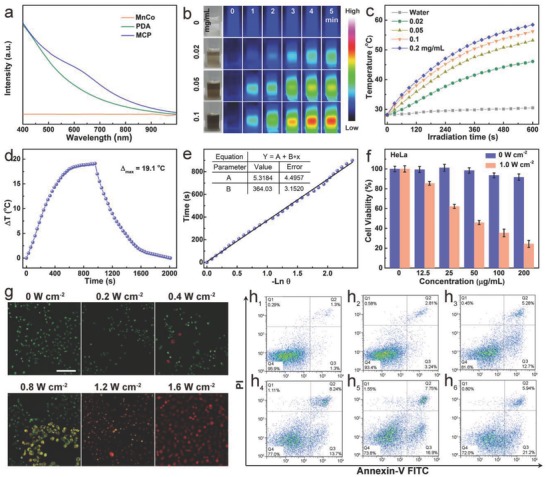
Photothermal properties and in vitro PTT of MCP NPs. a) UV–vis–NIR spectra of MOFs, PDA, and MCP NPs. b) Infrared thermal photographs of MCP aqueous solutions containing different concentrations captured before and after being irradiated using a 808 nm laser for 5 min (1.0 W cm^−2^). c) Temporal temperature evolutions of MCP solutions with various concentrations. d) Plot of temperature change (∆*T*) over a period of 900 s versus the concentration of MCP (0.05 mg mL^−1^). e) Linear time data versus −ln θ obtained from the cooling period of Figure 2d. f) Relative viabilities of HeLa cells incubated with MCP‐PEG at different concentrations with/without laser irradiation (808 nm, 1.0 W cm^−2^) for 5 min. g) Confocal fluorescence images of MCP‐PEG‐incubated (50 µg mL^−1^) HeLa cancer cells after being irradiated by 808 nm laser at different power densities for 5 min; the cells were co‐stained by calcein AM and propidium iodide before imaging. The scale bar is 150 µm. h1–h6) Flow cytometry analysis of HeLa cells incubated with MCP‐PEG with different power densities for 5 min. The four areas represent the different phases of the cells: necrotic (*Q*1), late‐stage apoptotic (*Q*2), early apoptotic (*Q*3), and liver (Q4).

A quantitative study of the photothermal conversion efficiency (η) of MCP at 50 µg mL^−1^ with a power density of 1.0 W cm^−2^ (Figure 2d,e) was carried out and calculated according to the previous work.^14^, ^21^ Results show that the η of MCP NPs was determined to be 41.3%, which is high enough for effective PTT compared with other materials in literatures such as polydopamine dots (38%), Cu_2−_ *_x_*Se (22%), and Au nanorods (21%) (Table S2, Supporting Information). Importantly, based on the observation in Figure 2a, MCP NPs showed a stronger NIR absorption peak at 808 nm compared with PDA NPs at the same PDA concentration. The result indicates that the MCP NPs may have higher photothermal conversion efficiency than PDA NPs. The calculated results show that the η of PDA NPs was calculated to be 36.9% (Figure S10, Supporting Information). Such an enhanced photothermal effect (the increased ratio: 11.8%) of MCP NPs may come from π–π stacking interactions between the polarizable π‐electron clouds of the —C≡N— linkers and PDA, which leads to the enhanced electron density in MCP NPs.

Next, we evaluated the in vitro cell toxicities of MnCo, MCP, and MCP‐PEG NPs (Figure S11a,b, Supporting Information). Results show that after being hybridized and modified by MOF NPs with biocompatible PDA and PEG, respectively, the resultant MCP and MCP‐PEG NPs demonstrate nontoxic behavior to HeLa and 4T1 cells during 24 h at a concentration up to 200 µg mL^−1^ in comparison to MnCo NPs. HeLa cells were chosen as a model to assess in vitro PTT ability of MCP‐PEG NPs. As shown in Figure 2f, HeLa cells treated by MCP‐PEG were efficiently killed in a concentration‐dependent manner under NIR laser irradiation (1.0 W cm^−2^, 5 min); nearly 70 ± 3.5% cells were dead under irradiation at a concentration of 200 µg mL^−1^. 4T1 cells indicated the similar vulnerability upon laser irradiation in the presence of MCP‐PEG (Figure S11c, Supporting Information). Plenty of green living cells with a few apoptotic and/or necrotic cells in the control can be detected from confocal images. With the increase of laser power from 0.2 to 1.6 W cm^−2^, an increased area of red‐stained cells can be observed, indicating that MCP‐PEG NPs can act as a heat mediator for hyperthermia treatment of cancer cells (Figure 2g). Furthermore, flow cytometry–based apoptosis analysis of annexin V‐FITC and propidium iodide (PI) double‐stained HeLa cells was performed, an obvious increased apoptotic rate (*Q*2 + *Q*3) is presented with the increase of laser power, indicating that the major PTT‐induced cell death type was apoptotic (Figure 2h_1_–h_6_).

MnCo is a well‐known double‐perovskite framework, in which the —C≡N— linkers are ordered to create high‐spin Mn–N_6_ (*S* = 5/2, five unpaired 3d electrons) octahedra. The MCP‐PEG NPs were selected to first assess in vitro *T*_1_ MR imaging function. The *T*_1_‐weighted MR images of MCP‐PEG NPs show a concentration‐dependent brightening effect under a 3T MR clinical scanner (**Figure** **3**a). The corresponding longitudinal relaxivity (*r*_1_) value of MCP‐PEG NPs was calculated to be 5.175 m m ^−1^ s^−1^ (Figure 3b), which is slightly larger than that of a commercially Gd‐based CAs (Magnevist, 4.25 mm ^−1^ s^−1^).^22^ MRI tests of HeLa cells incubated with MCP‐PEG, MCP‐PEG‐RGD NPs, and MCP‐PEG‐RGD + excess RGD (blocking) at the same Mn concentration (12.5 µg mL^−1^) for 24 h were performed (Figure 3c). Results show that the MCP‐PEG‐RGD NPs demonstrate more brightness signal than MCP‐PEG NPs and the blocking samples due to their enhanced cell uptake. Moreover, the concentrations of Mn in 2.5 million HeLa cells were determined by inductively coupled plasma mass spectrometry (ICP‐MS). The quantification results demonstrated that MCP‐PEG‐RGD had a significantly higher value, in consistent with the MRI results, which indicates the MCP‐PEG‐RGD NPs possess targeting ability for HeLa cells (Figure 3d,e).

**Figure 3:**
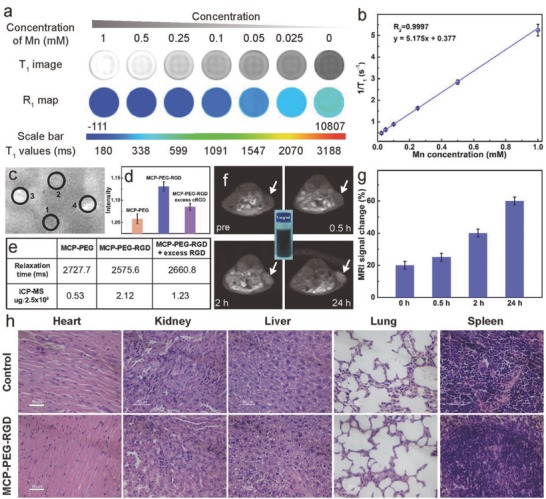
In vitro and in vivo MR imaging. a) *T*_1_‐weighted MR images and *R*_1_ map of MRI phantom images of MCP‐PEG NPs at different Mn concentrations. b) Plot of 1/*T*_1_ as a function of Mn concentration. c) *T*_1_‐weighted MR images of saline (1), MCP‐PEG (2), MCP‐PEG‐RGD NPs (3), and MCP‐PEG‐RGD + excess cRGD (4) incubated with HeLa cells for 24 h. d) The corresponding relative MRI intensity of (2), (3), and (4) incubated with HeLa cells. e) The corresponding relaxation time and intracellular Mn^2+^ content in 2.5 million HeLa cells. f) *T*_1_‐weighted MR images of HeLa tumor‐bearing mouse at different time postinjection of MCP‐PEG‐RGD NP injection (the inset is a photograph of aqueous MCP‐PEG‐RGD dispersion in a vial with a concentration of 1 mg mL^−1^). g) Quantification analysis of *T*_1_‐weighted MR signals in tumor sites. h) Slices of main organs stained with hematoxylin and eosin (all the scale bars are 50 µm).

To further evaluate the MR imaging in vivo, the MCP‐PEG‐RGD NPs were administered through intravenous (i.v.) injection at the dose of 5 mg kg^−1^. As shown in Figure 3f, *T*_1_‐weighted MR signals gradually show up in the tumor, indicating time‐dependent tumor accumulation of MCP‐PEG‐RGD NPs. Quantitative analysis further confirmed that the average MR signals in the tumor of mice, treated by MCP‐PEG‐RGD NPs, gradually increased over time (Figure 3g). All the animal experiments were performed following the university laboratory animal guidelines with approval from the Animal Care Committee of University of Science and Technology of China and the Ethical Committee of the Experimental Animal Center of Anhui Medical University. In addition to MR imaging, the fluorescent imaging function of MCP‐PEG‐RGD NPs was also measured by studying the photoluminescence property and in vitro fluorescence imaging ability. After being incubated with NPs, the HeLa cells became brightly illuminated in multicolor forms at different excitation wavelengths including single‐photon channels (λ_ex_ = 405, 488 nm), and two‐photon channels (λ_ex_ = 720, 750, and 780 nm) (Figure S12, Supporting Information).

Before in vivo therapeutic efficiency, the biocompatibility and biodistribution of MCP‐PEG‐RGD NPs were further assessed. The hemolysis test shows that no visible hemolytic effects (2%) were observed even at a MCP‐PEG‐RGD concentration up to 1 mg mL^−1^ in PBS, which indicates their excellent biocompatibility (Figure S13, Supporting Information). In addition, the half‐time (≈ 3 h) of MCP‐PEG‐RGD NPs is slightly longer than that of other MOFs without PEGylated in our previous report (≈2.65 h), and revealed that the PEG modification could indeed prolong the blood circulation time (Figure S14, Supporting Information).^23^ To further evaluate the biocompatibility, histological analysis of various tissues from mice treated with MCP‐PEG‐RGD or PBS solution (as control) was performed (Figure 3h). There was no appreciable organ damage or inflammation of the mice in both groups. The biodistribution of MCP‐PEG and MCP‐PEG‐RGD NPs in different organs was also studied (Figure S15, Supporting Information). Results showed that liver, kidney, and spleen have relatively high uptake of particles due to uptake by macrophage in reticuloendothelial systems. MCP‐PEG had slightly increased uptake in the liver (19.1 ± 1.9% ID g^−1^) compared with MCP‐PEG‐RGD (17.9 ± 2.6% ID g^−1^). Aside from liver, there were no big differences between other tested organs. Meanwhile, the tumor accumulations of MCP‐PEG‐RGD and MCP‐PEG NPs are ≈8.3% ID and ≈5.2% ID, respectively (Figure S16, Supporting Information), which indicates that the MCP‐PEG‐RGD NPs should have higher PTT efficiency in vivo due to the targeting ability of RGD molecules.

Motivated by the perfect in vitro PTT efficiency, in vivo biocompatibility, and high tumor accumulation, we next performed in vivo PTT ability of MCP‐PEG and MCP‐PEG‐RGD on HeLa tumor‐bearing mice. Four groups (*n* = 6) of Hela tumor‐bearing female nude mice were used. Compared with alone saline and alone NIR treated groups with uncontrolled growth of tumor within 14 d, other two treated groups, by MCP‐PEG + NIR and MCP‐PEG‐RGD + NIR, showed the inhibited tumor growth (**Figure** **4**a). The tumor sizes in alone saline and alone NIR groups were much larger than that of in the group treated with MCP‐PEG + NIR and MCP‐PEG‐RGD + NIR (Figure 4b). Remarkably, the ablated tumors became slightly black scars at the original sites. Besides, much enhanced inhibitory rate could be observed for the MCP‐PEG‐RGD group, which is attributed to the enhanced tumor target effect. In addition, all tumors were collected and weighed after post‐treatment at day 14, and the tumor mass was consistent with the tumor grown curves and tumor volume (Figure 4a,c). Furthermore, it is found that all groups showed the similar body increase tendency even after intravenous injection of MCP‐PEG or MCP‐PEG‐RGD (Figure 4d). No obvious weight loss was observed, indicating the good biocompatibility of MCP‐PEG or MCP‐PEG‐RGD.

**Figure 4:**
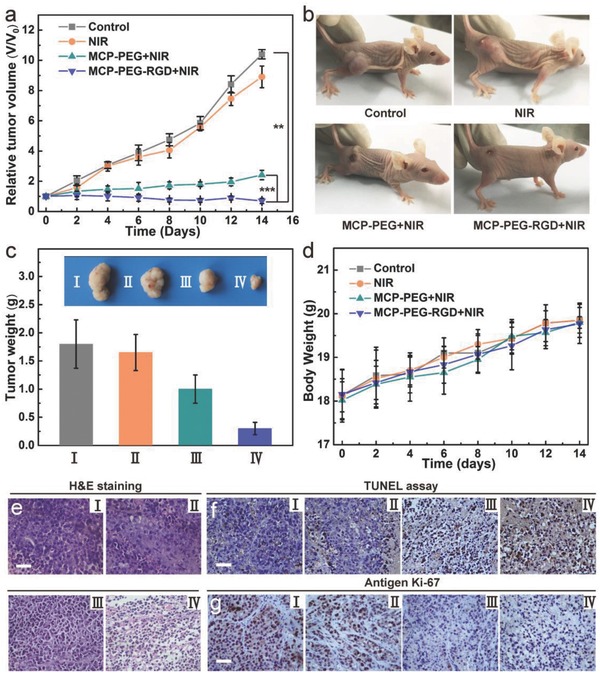
In vivo PTT. a) Tumor growth curves of mice after different treatments (*n* = 6). b) Representative photographs of mice at various different treatments for 14 d at indicated taken after treatment. c) Average weights and typical photographs (inset) of tumors collected from mice at the end of treatments (day 14). d) Average body weights of mice after different treatments indicated. Optical microscopy images of tumor sections stained by e) hematoxylin and eosin (H&E), f) TdT‐mediated dUTP nick‐end labeling (TUNEL) and g) Ki‐67 from the groups of (I) control, (II) NIR, (III) MCP‐PEG + NIR, and (IV) MCP‐PEG‐RGD + NIR. Statistical analysis was performed using the Student's two‐tailed *t*‐test (** *p* < 0.01 and *** *p* < 0.001).

To further evaluate the possible side effect, hematoxylin and eosin (H&E) stained slices of tumors of the four groups were performed (Figure 4e). There was no appreciable damage or inflammation of the mice in the control and NIR groups, while prominent cell damage in the tumors of the other two groups (MCP‐PEG+NIR and MCP‐PEG‐RGD+NIR) was found because the tumor site is treated by NIR irradiation. Next, the terminal deoxynucleotidyl transferase (TdT)‐mediated deoxyuridine triphosphate (dUTP) nick end labeling (TUNEL) staining and Ki‐67 assay were employed for tumor slices collected post various treatments to determine tumor cell apoptosis and proliferation levels, respectively (Figure 4f,g). As expected, tumor slices from MCP‐PEG‐RGD + NIR group showed the highest level of TUNEL positive signals, which were much lower in control group, alone NIR group, and in MCP‐PEG + NIR group. The cell proliferation was assessed by Ki‐67 as Ki‐67 can stain the proliferative cells into brown. From the Ki‐67 assay result, it can be found that the MCP‐PEG‐RGD + NIR group keeps the least brown cells and exerts an excellent inhibiting effect on cell proliferation.

In summary, we have developed a simple, one‐pot process for in situ hybridization of dopamine in the skeleton of MOFs. Through accommodating PDA in pores of MOFs, the resulting MCP NPs can not only be served as a positive *T*_1_ MR contrast agent, but also demonstrate an enhanced photothermal conversion capacity for cancer therapy. After the further PEGylation and targeting modification, the obtained MCP‐PEG‐RGD NPs are featured with a number of unique advantages over commonly explored photothermal agents: uniform size distribution, long‐term solution stability, enhanced photothermal conversion efficiency, and higher tumor accumulation. Combined with the additional functions including *T*_1_ MR imaging, fluorescent imaging, and tumor targeting, these nanoscale MOF–polymer hybrid nanogels will be highly promising for the development of multiplex theranostic nanoplatforms.

## Conflict of Interest

The authors declare no conflict of interest.

## Supporting information

Supplementary
