# Supplementary material for: In Situ One‐Pot Synthesis of MOF–Polydopamine Hybrid Nanogels with Enhanced Photothermal Effect for Targeted Cancer Therapy
Source: Adv Sci (Weinh). 2018 Apr 14;5(6):1800287. doi: 10.1002/advs.201800287 (PMC6010715; doi:10.1002/advs.201800287)
Supplement: Supplementary file 1 — Supplementary [file ADVS-5-1800287-s001.pdf]

Copyright WILEY-VCH Verlag GmbH & Co. KGaA, 69469 Weinheim, Germany, 2018.

ADVANCED SCIENCE
Open Access

Supporting Information

for *Adv. Sci.,* DOI: 10.1002/advs.201800287

In Situ One-Pot Synthesis of MOF–Polydopamine Hybrid Nanogels with Enhanced Photothermal Effect for Targeted Cancer Therapy

Dongdong Wang, Huihui Wu, Jiajia Zhou, Pengping Xu, Changlai Wang, Ruohong Shi, Haibao Wang, Hui Wang, Zhen Guo,* and Qianwang Chen**

Copyright WILEY-VCH Verlag GmbH & Co. KGaA, 69469 Weinheim, Germany, 2013.

Supporting Information

for *Adv. Mater.*, DOI: 10.1002/advs2018000287

In Situ One-Pot Synthesis of MOFs-Polydopamine Hybrid Nanogels with Enhanced Photothermal Effect for Targeted Cancer Therapy

Dongdong Wang, Huihui Wu, Jiajia Zhou, Pengping Xu, Changlai Wang, R. Shi, Haibao Wang, Hui Wang*, Zhen Guo*, Qianwang Chen*

D. Wang, P. Xu, C. Wang, R. Shi, Prof. H. Wang, Prof. Q. Chen
Hefei National Laboratory for Physical Sciences at Microscale
Department of Materials Science &Engineering
University of Science and Technology of China
High Magnetic Field Laboratory
Hefei Institutes of Physical Science, Chinese Academy of Sciences
Hefei 230026, P. R. China
E-mail: cqw@ustc.edu.cn

H. Wu, Dr. J. Zhou, Prof. Z. Guo
School of Life Sciences
University of Science and Technology of China
Hefei 230026, P. R. China
E-mail: zhenguo@ustc.edu.cn

Prof. H. Wang
Department of Radiology
First Affiliated Hospital of Anhui Medical University
Hefei, 230022, P. R. China

*Corresponding Author: hw39@hmfl.ac.cn; zhenguo@ustc.edu.cn; cqw@ustc.edu.cn

Experimental Section

Materials: Dopamine, potassium cobaltocyanide ($K_3[Co(CN)_6]$) were obtained from Alfa Aesar. Manganese acetate ($Mn(COO)_2·4H_2O$), poly(vinylpyrrolidone) (PVP, K-30) were obtained from Sinopharm Group Co. Ltd. Mal-PEG-SCM (M_w= 5000) was obtained from Creative PEG works. Cyclo(Arg-Gly-Asp-D-Phe-Cys) (cRGD-Cys, RGD-SH) was purchased from NJPeptide Inc. Other chemicals were obtained from Sinopharm Group Co. Ltd. All chemicals were used as received without further purification.

Synthesis of MnCo, MCP, PDA and MOFs-PDA intermediate: The synthesis of MnCo is realized through a coprecipitation method as previously reported by our group with a modification.[1] For synthesis of MCP, 18.1 mg $Mn(COO)_2·4H_2O$ and 20 mg dopamine were added to 15/5 ml ethanol/water solution and stirred for 30 min under pH 8.5. After that small coordination polymer of dopamine-Mn^{2+} were obtained. Then 10 g of a solution containing 16.6 mg of $K_3[Co(CN)_6]$ was added dropwisely. After being stirred for 24 h, excess unreacted dopamine molecules were removed by centrifugation under 12000 rpm for 20 min for three times with a mixture of ethanol and H_2O. The synthesis of PDA is similar to that of MCP without adding $Mn(COO)_2·4H_2O$ and $K_3[Co(CN)_6]$. The MOFs-PDA intermediate is synthesized through stopping the reaction one hour later during the synthesis of MCP.

Conjugation of MCP with PEG and cRGD (MCP-PEG, MCP-PEG-RGD): The resulted MCP nanoparticles with abundant -NH_2 on the surface were redispersed in 20 ml pure water. Afterward, 50 mg of Mal-PEG-SCM were added and reacted for another 1 h, resulting in MCP-PEG-Mal after purification by centrifugation. The cRGD-SH conjugation onto the NPs was achieved by a reaction between the maleimide group of the PEG conjugated on the surface of MCP nanoparticles and the thiol group of the cRGD. In a typical synthesis process, 1 mL portion of MCP-PEG-Mal solution was mixed with 0.5 mL of 1.5 mg mL^{-1} of cRGD peptide solution (the molar ratio of original added maleimide to cRGD peptide is 1:2) and gently stirred for 3 h at room temperature. Next, the product was further washed with PBS for

three times and purified through dialysis (M_w = 100 kDa) against deionized water for 2 days. The resulted MCP-PEG-RGD was redispersed with PBS buffer for further use.

Cellular uptake and Two-photon Fluorescence Analysis: HeLa cells were seeded on Chambered cover glass (Lab-Tek Chambered 1.0 Borosilicate Cover Glass system, Nunc). After 24 h, the cells were cultured to 50-60% confluency, and incubated with MCP-PEG-RGD at 37 °C under 10% CO_2 atmosphere for 24 h. Then the cells were washed with DMEM medium for three times, and replaced the medium by CO_2-independent medium (Gibco) containing 10% (vol/vol) fetal bovine serum (Hyclone, Logan, UT). Subsequent image analysis was taken by CLSM (Zeiss LSM 710).

In Vitro Cellular Toxicity Test: HeLa human cervical cancer cells and 4T1 murine breast cancer cells were originally obtained from American Type Culture Collection (ATCC). Human cervical carcinoma (HeLa) cells were cultured in 96-well plates at 1×10^4 cells per well and incubated in 5% CO_2 at 37 ºC for 24 h. Then different concentrations of MnCo and MCP-PEG (12.5, 25, 50, 100, and 200 μg mL^{-1}) were added. After that, the cells were further incubated for 24 h, and excess unbound materials were washed for three times with PBS. Subsequently, the relative cell viabilities (%) were detected by the standard MTT assay.

For in vitro photothermal therapy, HeLa and 4T1 cancer cells were seeded into 96-well plates and then incubated with various concentrations of MCP−PEG at 37 °C. Then the cells were exposed to an 808 nm NIR laser at the power density of 1.0 W cm^{-2} for 5 min. The relative cell viabilities after photothermal ablation were measured using the standard MTT assay. For imaging, HeLa cancer cells incubated with MCP-PEG (50 μg mL^{-1}) after irradiation by the 808 nm laser at various power density of 0, 0.2, 0.4, 0.8, 1.2, 1.6 W cm^{-2} for 5 min were stained by calcein AM and PI and then imaged using a microscope. The apoptosis and necrosis induced by photothermal toxicity were evaluated by flow cytometry. HeLa cells (1×10^4) were seeded into a 12-well plate and incubated with MCP-PEG (50 μg mL^{-1}). After irradiation by the 808 nm laser at various power density of 0, 0.2, 0.4, 0.8, 1.2, 1.6 W cm^{-2} for

5 min, the cells were harvested, detected, and quantified by apoptosis by an annexin V-FITC/PI apoptosis detection kit using a Guava EasyCyte 6-2L flow cytometer.

Magnetic Resonance Imaging (MRI) Measurement: For MRI test: MCP-PEG nanoparticles at various Mn concentrations (0, 0.025, 0.05, 0.1, 0.25, 0.5, and 1.0 mM) were measured at 25 ℃ with a clinical magnetic resonance (MR) scanner (GE HDxt, 3.0 T). For *in vivo* MRI imaging, we conducted MRI of tumor-bearing female BALB/c nude mice with an average body weight of 18 g (Shanghai SLAC Laboratory animal Co., Ltd.,). All the animal experiments were performed following the university laboratory animal guidelines with approval from the Animal Care Committee of University of Science and Technology of China and the Ethical Committee of the Experimental Animal Center of Anhui Medical University. Before injection of MCP-PEG-RGD (1.0 mg/mL in PBS, 5 mg of per kilogram of mouse body weight) via the tail vein, mice were anesthetized intraperitoneal injection of 10% chloral hydrate (3.0 g per kg body weight). T_1-weighted MR imaging were acquired at pre-injection, 30 min, 2 h and 24 h post-injection of MCP-PEG-RGD. T_1-weighted MR images were acquired using fast spin echo multislice (fSEMS) sequence with parameters: TR/TE = 780/19.6 ms, number of excitations = 2, echo train length = 2, 0.188 × 0.188 mm in plane resolution with a slice thickness of 2 mm.

Cell uptake study of MCP-PEG-RGD and in vitro MRI: HeLa cells were seeded on Chambered cover glass (Lab-Tek Chambered 1.0 Borosilicate Cover Glass system, Nunc) until (~2.5×10^6 cells per flask) was observed. MCP-PEG and MCP-PEG-RGD at a Mn concentration of 12.5 μg mL^{-1} in DMEM was added to the culture dishes and incubated with HeLa cells at 37 ^oC under 10% CO_2 atmosphere for 24 h. For the blocking experiment, HeLa cells were pretreated with 20 mM free RGD for 30 min prior to adding MCP-PEG-RGD. After incubation, the cells were washed with PBS three time and collected for MRI test. The total Mn content in those cells was measured by ICP-MS.

Blood Circulation and Biodistribution: To determine the blood circulation of MCP-PEG-RGD, NPs were injected into the tail vein at dose of 5 mg/kg (n = 3). At the predetermined time points, 10 μL blood was extracted from the tail and then digested with 0.1 mL aqua regia (HCl / HNO_3 = 3:1) before analyses of Mn contents using ICP-AES. To determine the biodistribution of our NPs, major organs and tissues (tumor, liver, spleen, lung, kidney, intestine, stomach, and heart) from HeLa-tumor-bearing mice (n= 3) were collected at the indicated time point. Next, the collected organs were wet-weighted and digested with aqua regia under heating to boiling for 2 h, and Mn contents in tissues were analyzed using ICP-AES.

In vivo Photothermal Therapy: Once palpable tumors in BALB/c nude mice were established for xenograft experiments, they were randomly allocated into four groups (control, NIR, MCP-PEG+NIR, MCP-PEG-RGD+NIR, n=5). MCP-PEG and MCP-PEG-RGD in Saline buffer (total dose=100 μL, $C_{[MCP\text{-}PEG]}$=1.0 mg mL^{-1}) were injected via the tail vein, while mice in the control group were only injected with saline (100 μL). After 24 h, the tumors from group 2, 3 and 4 were irradiated with an 808 nm laser (1.0 W cm^{-2}, 5 min). Tumor dimensions were measured with a caliper every 2 days after drug administration, and the tumor volume was calculated according to the equation: Volume=(Tumor length)×$(\text{Tumor width})^2/2$ (mm^3). Relative tumor volume was normalized to its initial size before administration and laser irradiation. All mice were sacrificed after experiment and tumors of each group were collected.

Pathological Investigation: The tumor tissues and main organs of the administrated mice were resected, fixed in 4% formalin and then embedded in paraffin blocks. Formalin-fixed paraffin-embedded tissue samples were sectioned, stained with hematoxylin and eosin (H&E) and then examined with microscope. For Ki-67 and staining, tumor sections were incubated with the anti-Ki-67 rabbit polyclonal antibody (Abcam, ab15580, America) overnight at 4 ºC. And terminal deoxynucleotidyl transferase dUTP nick end labeling (TUNEL) staining was

performed using in situ cell death detection kit (POD, Roche, America). All these process were followed the manufacturer's guidelines. Then, Olympus IX-70 microscope was used to acquire images of the stained tumor and organ slices.

Characterization: Transmission electron microscopy (TEM) images were obtained from a transmission electron microscope (Hitachi H-7650) with an accelerating voltage of 100 kV. Powder X-ray diffraction (PXRD) patterns were performed on a Japan Rigaku D/MAX-cAX-ray diffractometer equipped with Cu Ka radiation. High-angle annular dark field (HAADF)-STEM and energy-dispersive X-ray (EDX) analyses were performed on a JEOL ARM-200F field-emission transmission electron microscope operating at 200 kV accelerating voltage. Scanning electron microscopy (SEM) images were acquired on a JEOL JSM-6700M microscopy. The content of Mn and Co were measured by inductively coupled plasma-atomic emission spectrometer mass spectrometer (ICP-MS) (Optima 7300DV). The photoluminscence (PL) spectra measurements were measured using a JY Fluorolog-3-Tou fl uorescence spectrophotometer equipped with a 450 W Xe light source and double excitation monochromators. Ultraviolet-visible (UV-Vis) absorption spectra were measured on a SOLID3700 spectrometer. The dynamic laser light scattering (DLS) measurements were performed with a Zetasizer UV spectrometer (Malvern μV).

Supplementary Figures

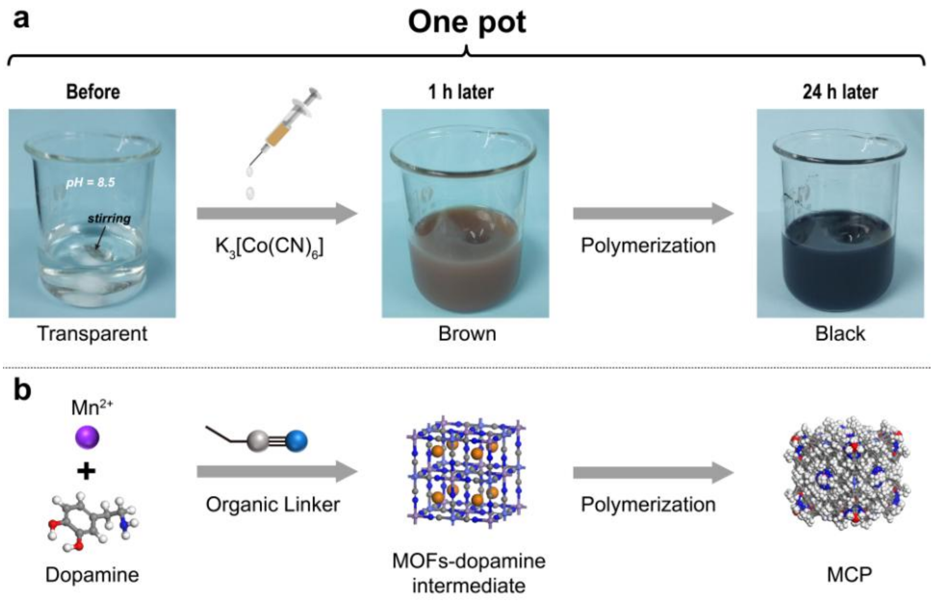

Figure S1. (a) The color change of the reaction solution during the whole procedure. (b) The corresponding scheme.

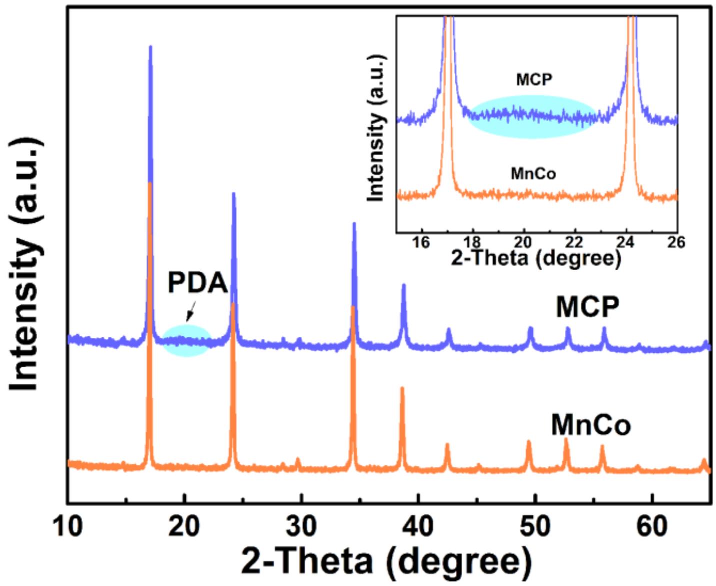

Figure S2. The PXRD of MOFs and MCP (inset is the enlarged part of 2-Theta from 15-26 degree).

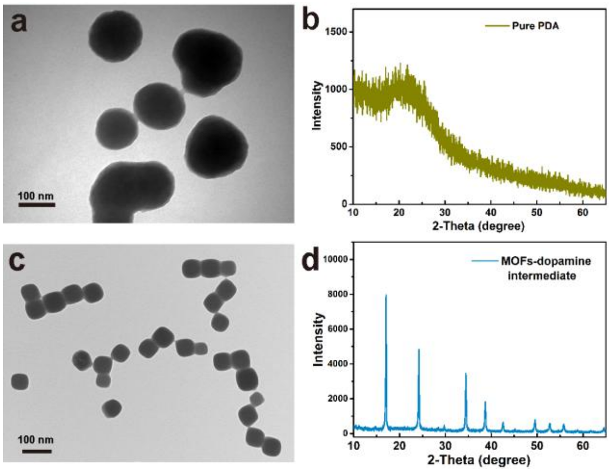

Figure S3. a) and c) TEM images of PDA and MOFs-dopamine intermediate, respectively. b) and d) The PXRD of PDA and MOFs-dopamine intermediate, respectively.

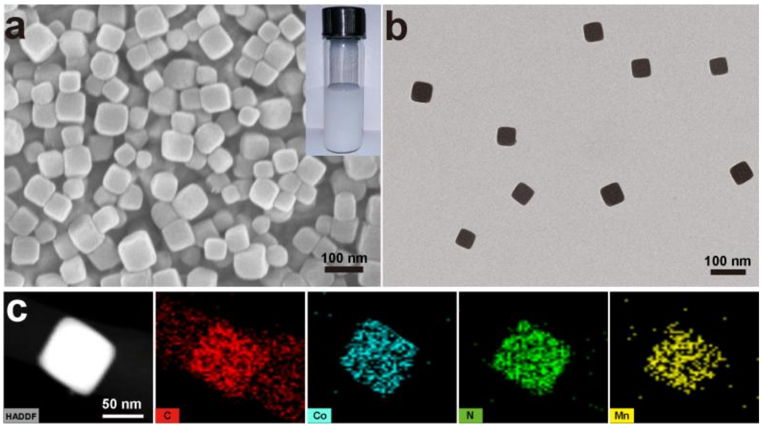

Figure S4. a) SEM image, b) TEM image and c) HAADF-STEM image of MnCo MOFs. The inset in a is a photograph of aqueous MnCo dispersion in a vial.

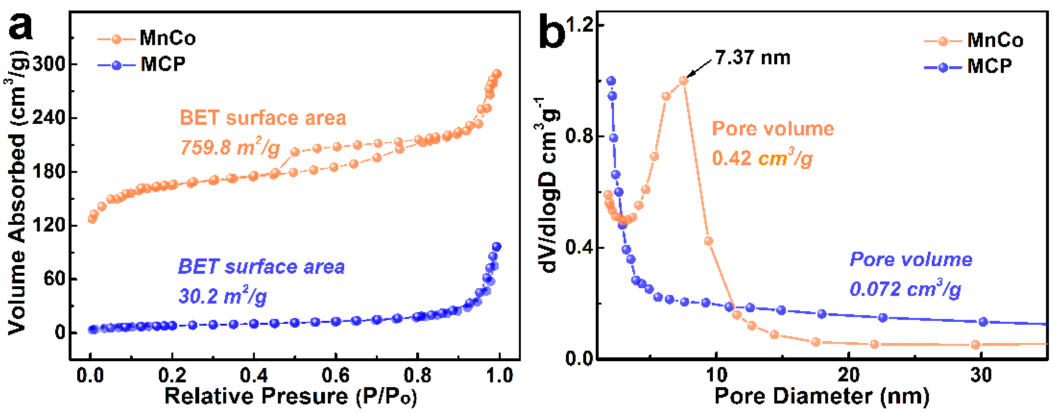

8

Figure S5. (a) Nitrogen adsorption-desorption isotherms curves and (b) Pore size distribution of MnCo and MCP.

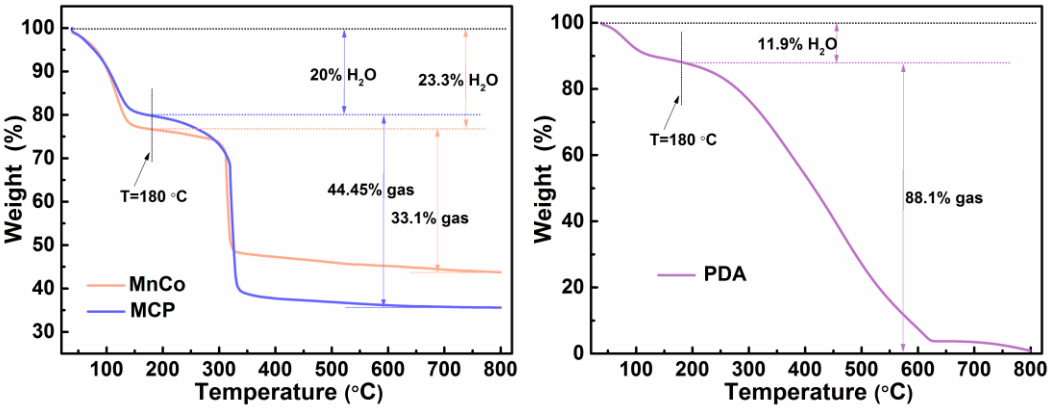

Figure S6. Thermogravimetric analysis of MnCo, MCP and PDA.

Thermogravimetric analysis (TGA):

TGA of pure PDA, MnCo and MCP were also preformed to confirm the encapsulation capacity of PDA.

Table S1.

wt % Item	H_2O	Gas	remnant	Gas /(Gas+remnant)	remnant /(Gas+remnant)
**PDA**	11.9	88.1	0	100	0
**MCP**	20	44.45	35.55	56.8	43.2
**MnCo**	23.3	33.1	43.6	43.2	56.8

Assuming that the weight of PDA is x g and MnCo is y g (MCP is x+y=100 g).

100%x+43.2%y=56.8 g…………………①

56.8%y=43.2 g…………………………..②

So the value of x is 24 g and y is 76 g.

The weight ratio of MnCo/PDA in MCP was estimated to be 76/24 based on thermogravimetric analysis.

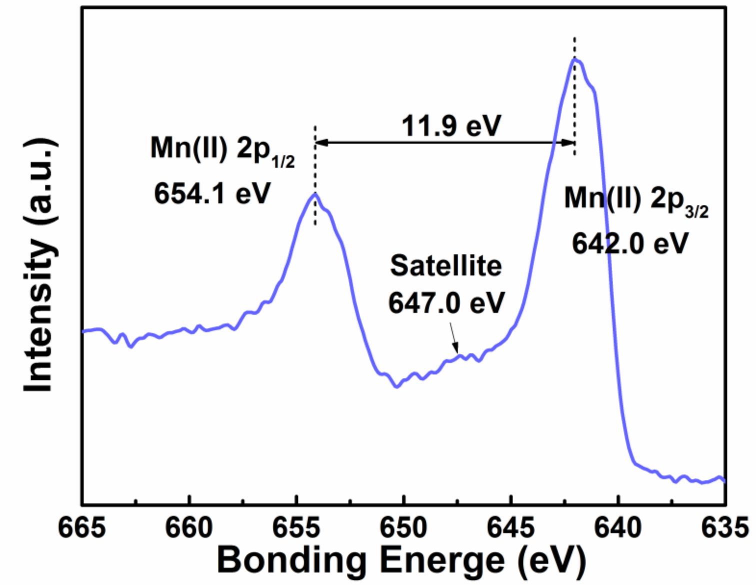

Figure S7. XPS spectrum of MCP NPs.

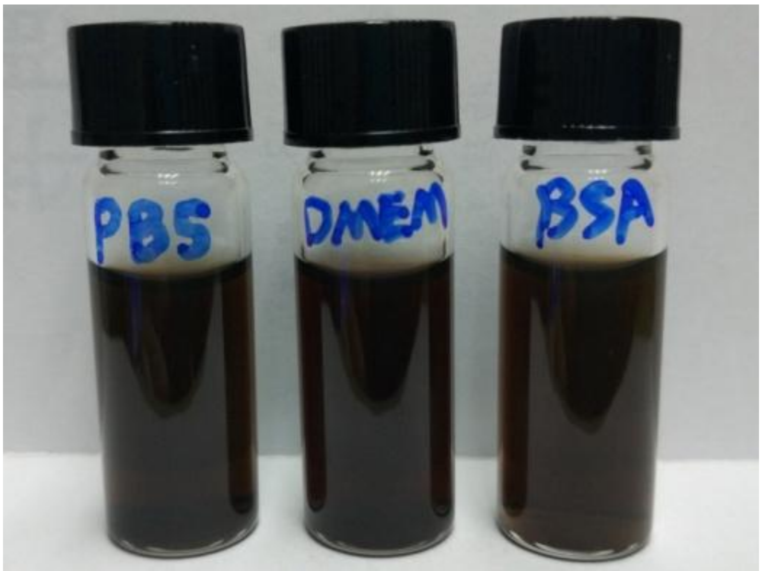

Figure S8. Colloidal dispersion of MCP-PEG in PBS, DMEM cell medium, and BSA.

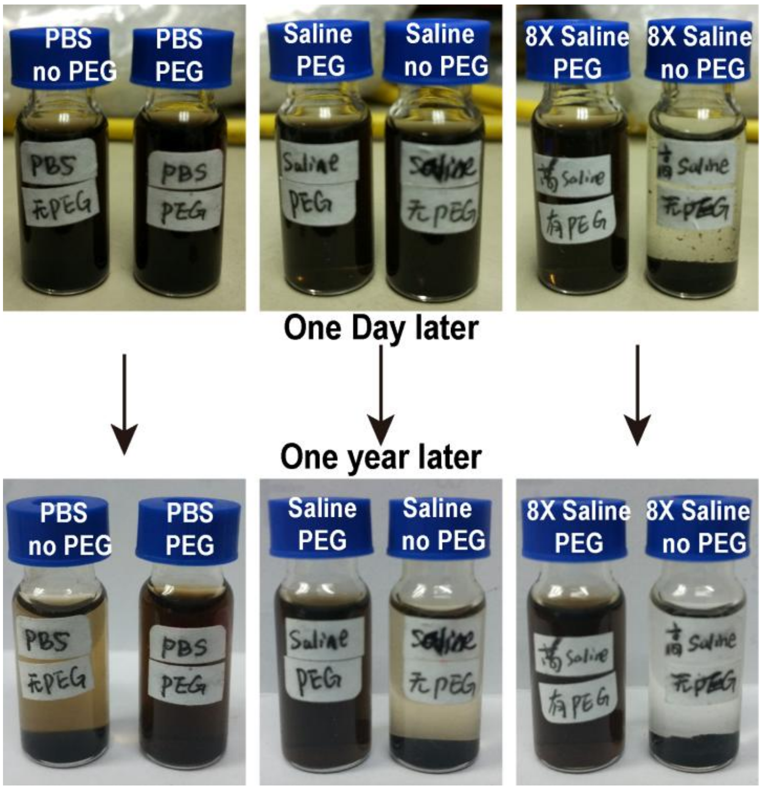

Figure S9. Colloidal stability of MCP and MCP-PEG NPs in PBS, 0.9% saline, and 7.2% saline (8x saline) for one day and one year.

Calculation of photothermal conversion efficiency:

The photothermal conversion efficiency of MCP-PEG and PDA-PEG were calculated using the model described in Roper's et al.[2] where the photothermal conversion efficiency is described by the following equations:

$\sum_i m_i C_{p,i} \frac{dT}{dt} = Q_{NPs} + Q_{diss} - Q_{loss}$ --------------------------(1)

where m and C_p are the mass and heat capacity of solvent (water), respectively. T is the solution temperature. Q_{NPs} is the photothermal energy input by nanoparticle. Q_{diss} is the heat associated with the light absorbance of the solvent. Q_{loss} is thermal energy lost to the surroundings.

$Q_{NPs} = I(1 - 10^{-A_{808}})\eta$ ---------------------------(2)

where η represents the photothermal conversion efficiency. A_{808} is the absorbance intensity of nanoparticle at 808 nm. I is the power density of laser (1.0 W/cm^2).

$Q_{loss} = hS\Delta T$ ----------------------------(3)

where h is the heat transfer coefficient. S is the surface area of the container. ΔT is the temperature change of the solution from that of the surroundings.

When the temperature of system reaching a steady stage, dT/dt in equation (1) is 0. Then, $Q_{NPs} + Q_{diss} = Q_{loss} = hS\Delta T_{max}$, and we get equation (4).

$\eta = \frac{hS\Delta T_{max} - Q_{diss}}{I(1-10^{-A_{808}})}$ ----------------------------(4)

To calculate hS: We herein introduce $\theta = \Delta T/\Delta T_{max}$, which is defined as the ratio of ΔT to ΔT_{max}. Then equation (1) will be change to equation (5).

$\frac{d\theta}{dt} = \frac{hS}{\sum_i m_i C_{p,i}}\left[\frac{Q_{NPs} + Q_{diss}}{hS\Delta T_{max}} - \theta\right]$ ----------------------------(5)

When the laser was shut off (the cooling stage), the $Q_{NPs}+Q_{diss} = 0$, equation (5) will be changed to equation (6).

$t = -\frac{\sum_i m_i C_{p,i}}{hS}\ln\theta$ ----------------------------(6)

hS can be determined by plotting time as a function of $-\ln\theta$ for the cooling curves (after switching off the laser) and inputting appropriate values for m (1 g) and C_p (4.2 J/g/ºC).

Q_{diss} is measured independently to be 0.0389 J/s.

For MCP (Figure 2d,e in manuscript): Where ΔT_{max} is 19.1 ºC, Q_{diss} is measured independently to be 0.0246 J/s, I is 1.0 W/cm^2 and A_{808} is 0.279. According to the linear time data versus $-\ln\theta$, and inputting appropriate values for m (1 g) and C_p (4.2 J/g/ºC) the photothermal conversion efficiency of MCP was calculated to be ~41.3 %. Besides, photothermal conversion efficiency of MCP NPs under different laser power and mass concentration was also studied.

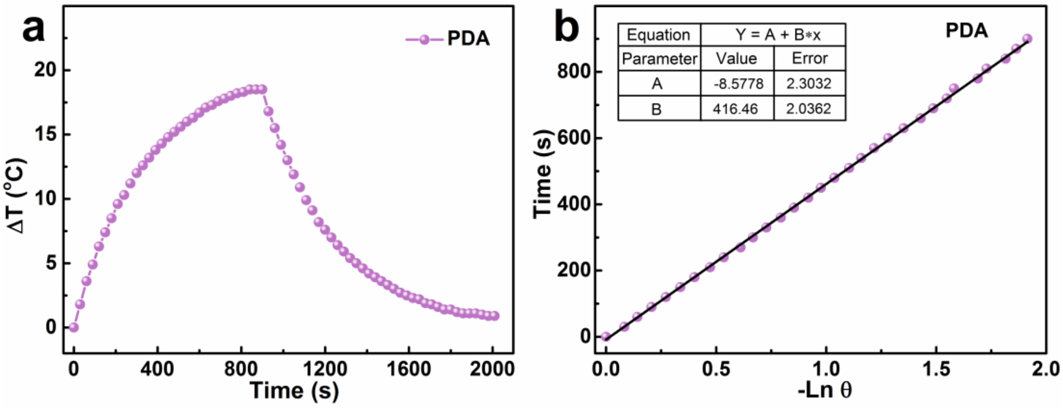

Figure S10. (a) The photothermal response of pure PDA aqueous solution for 900 s with an NIR laser (808 nm, 1 W cm^{-2}) and then the laser was shut off. (b) shows the linear time data versus -lnθ cooling period.

For pure PDA: Where ΔT_{max} is 18.5 ºC, Q_{diss} is measured independently to be 0.0246 J/s, I is 1.0 W/cm^2 and A_{808} is 0.251. According to the linear time data versus –lnθ, and inputting appropriate values for m (1 g) and C_p (4.2 J/g/ºC) the photothermal conversion efficiency of pure PDA was calculated to be ~36.9 %.

Table S2.

Materials	Mass (μg ml^{-1})	Laser density (W cm^{-2})	Wavelength (nm)	η (%)	Ref.
**MCP-PEG**	50	1.0	808	41.3	This work
**MCP-PEG**	75	1.5	808	42.1	This work
**MCP-PEG**	100	2.0	808	40.9	This work
**MCP-PEG**	200	2.0	808	41.8	This work
**UiO-66@PAN**	100	1.5	808	30.2	3
**Dopamine-Melanin**	200	2.0	808	40	4
**Dopamine CDs**	50	1.5	808	38	5

**Polypyrrole NPs**	20	1.0	808	44	6
**Carbon dots**	100	2.0	671	38.5	7
**Carbon nanospheres**	50	2.0	808	33	8
**Prussian Blue@ZIF-8**	100	1.6	808	22.9	9
**Au nanorods**	200	2.0	808	21	4
**Cu_9S_5**	40	0.51	980	25.7	10
**$Cu_{2-x}Se$**	50	2.0	800	22	11
**PVP-Bi nanodots**	62.5	1.3	808	30	12

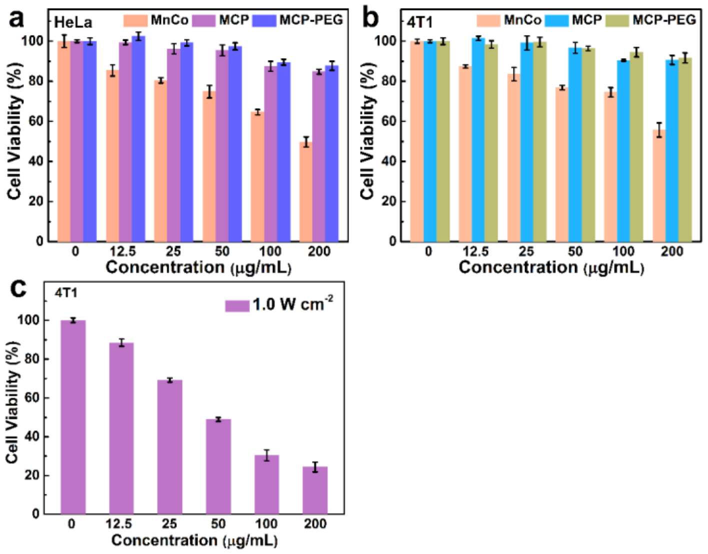

Figure S11. Relative viabilities of (a) HeLa cells and (b) 4T1 cells incubated with MnCo, MCP and MCP-PEG at various concentrations. (c) Relative viabilities of 4T1 cells incubated with MCP-PEG at different concentrations with laser irradiation (808 nm, 1.0 W cm^{-2}) for 5 min.

 14

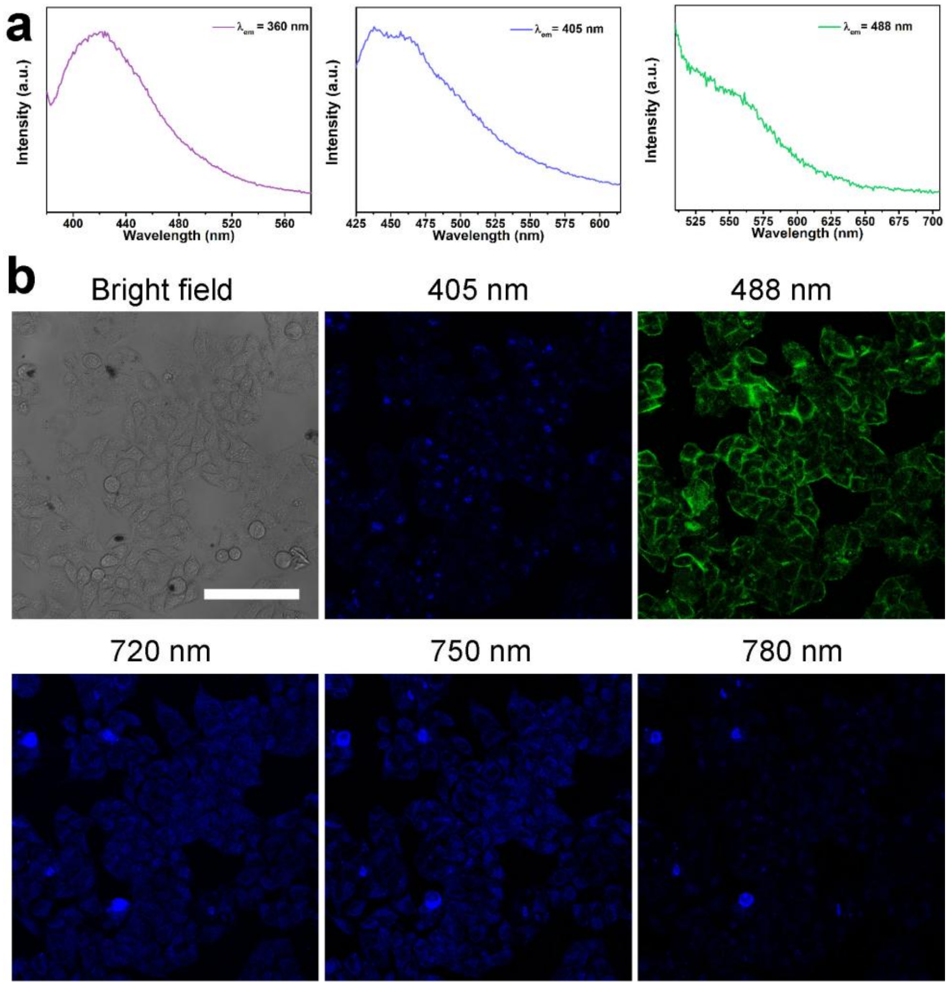

Figure S12. CLSM images of HeLa cells under 405-, 488 nm single-photon and 720-, 750-, and 780 nm two-photon excitation after incubation with MCP-PEG-RGD. Scale bar = 100 nm.

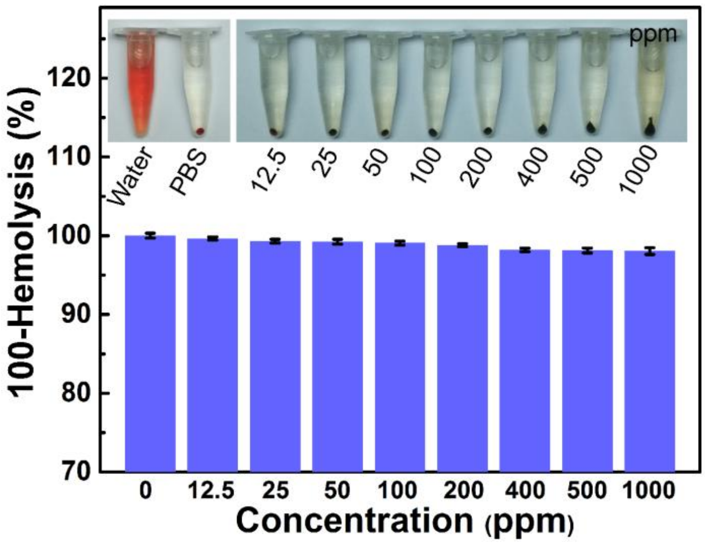

Figure S13. Hemolysis of MCP-PEG solution at various concentrations. The mixtures were centrifuged to detect the presence of hemoglobin in the supernatant visually (inset).

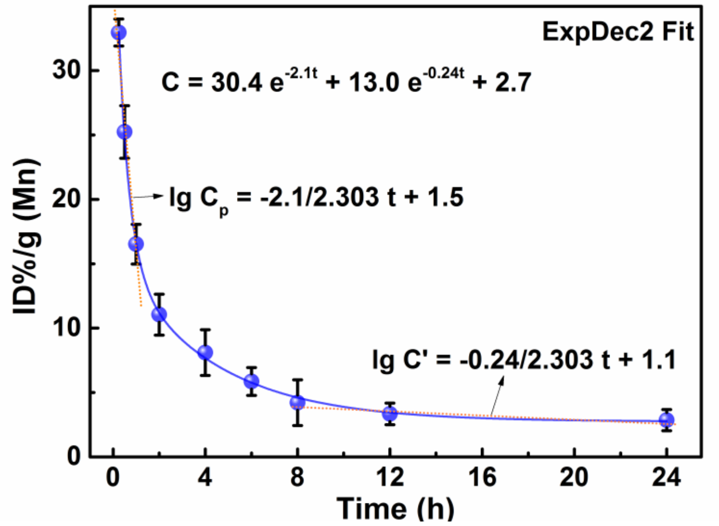

Figure S14. Blood circulation of MCP-PEG-RGD and the pharmacokinetics followed the two-compartment model.

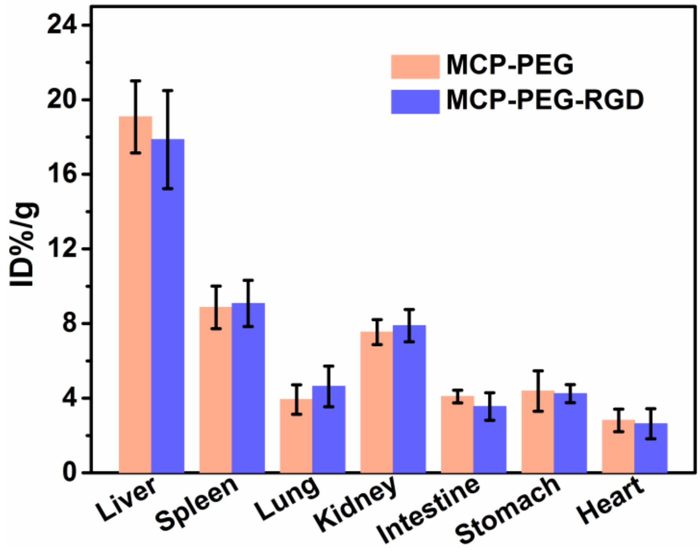

Figure S15. Bio-distribution of MCP–PEG-RGD in main organs of HeLa-tumor-bearing mice at 24 h post i.v. injection.

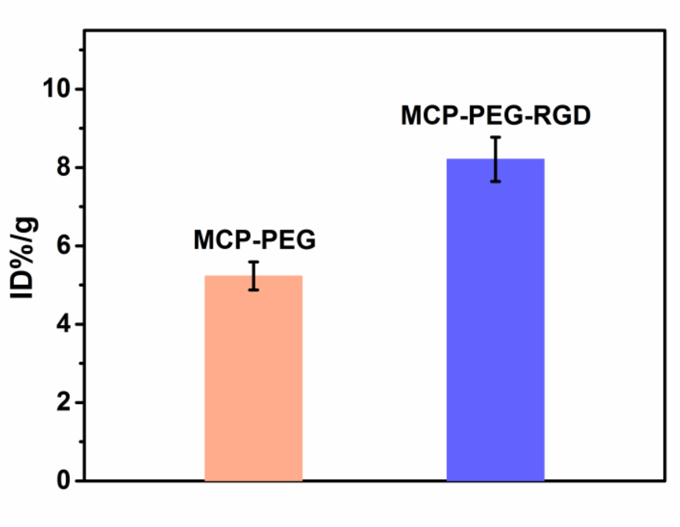

Figure S16. Tumor accumulation of MCP-PEG and MCP–PEG-RGD of HeLa-tumor-bearing mice at 24 h post i.v. injection.

Reference:

[1] L. Hu, P. Zhang, Q. W. Chen, N. Yan, J. Y. Mei, *Dalton Trans*. **2011**, *40*, 5557.
[2] D. K. Roper, W. Ahn, M. Hoepfner, *J. Phys. Chem. C* **2007**, *111*, 3636.
[3] W. Wang, L. Wang, Y. Li, S. Liu, Z. Xie, X. Jing, *Adv. Mater*. **2016**, *28*, 9320.
[4] Y. L. Liu, K. L. Ai, J. H. Liu, M. Deng, Y. Y. He, L. H. Lu, *Adv. Mater*. **2013**, *25*, 1353.
[5] Y. Li, X. Zhang, M. Zheng, S. Liu, Z. Xie, *RSC Adv*. **2016**, *6*, 54087.
[6] M. Chen, X. Fang, S. Tang, N. Zheng, *Chem. Commun*. **2012**, *48*, 8934.
[7] Z. Sun, H. Xie, S. Tang, X.-F. Yu, Z. Guo, J. Shao, H. Zhang, H. Huang, H. Wang, P. K. Chu, *Angew. Chem., Int. Ed.* 2015, 127, 11688.
[8] S. Wang, L. Shang, L. Li, Y. Yu, C. Chi, K. Wang, J. Zhang, R. Shi, H. Shen, G. I. Waterhouse, S. Liu, J. Tian, T. Zhang, H. Liu, *Adv. Mater*. **2016**, *28*, 8379.
[9] D. Wang, J. Zhou, R. Shi, H. Wu, R. Chen, B. Duan, G. Xia, P. Xu, H. Wang, S. Zhou, C. Wang, H. Wang, Z. Guo, Q. Chen, *Theranostics* **2017**, *7*, 4605.
[10] H. Lin, X. Wang, L. Yu, Y. Chen, J. Shi, *Nano Lett.* 2017, 17, 384.
[11] C. M. Hessel, V. P. Pattani, M. Rasch, M. G. Panthani, B. Koo, J. W. Tunnell, B. A. Korgel, *Nano Lett*. **2011**, *11*, 2560.
[12] P. P. Lei, R. An, P. Zhang, S. Yao, S. Y. Song, L. L. Dong, X. Xu, K. M. Du, J. Feng, H. J. Zhang, *Adv. Funct. Mater*. **2017**, *27*, 1702018.

17
